# Scoping Review of Outcome Measures Used in Lived Experience‐Led Mental Health Services

**DOI:** 10.1111/hex.70849

**Published:** 2026-09-07

**Authors:** Helena Roennfeldt, Heather Craig, Edith Botchway, Richard Schweizer, Christina Ng, Laura Hayes

**Affiliations:** ^1^ Mind Australia Burnley Australia; ^2^ One Door Mental Health Parramatta Australia

**Keywords:** experience measures, lived experience, outcome measures, peer‐run services, service evaluation

## Abstract

**Introduction:**

Service users have long advocated for alternative services led by people with firsthand or lived experience of mental health challenges. While several lived experience‐led services, such as peer respites, are available, their evaluations rarely incorporate outcome measures to assess impact, thereby limiting evidence of their validity as alternatives to clinical services. The aim of this review was to identify and describe the outcome and experience measures used in lived experience‐led services to better understand how evidence of their viability and acceptability is captured, and how this evidence supports these services as alternatives to traditional services.

**Methods:**

A scoping review of peer‐reviewed literature on lived experience‐led services was conducted by searching four databases (PsycINFO, Ovid Medline, CINAHL and Embase). Eligibility criteria included studies describing services led and delivered by people with lived experience (LE) of mental health challenges that implemented outcome or experience measures, and that focused on services outside clinical settings and not co‐delivered by traditional mental health professionals.

**Results:**

Thirteen studies were included that used a range of measures, including existing or adapted clinical and recovery survey outcomes. Results identify diversity in the outcomes measured and a lack of measures developed by the lived experience workforce.

**Conclusion:**

While the results highlight positive outcomes of lived experience run services, they also reveal a reliance on traditional outcome measures and a critical need to develop LE‐led outcome measures that align with lived‐experience values and practice.

**Lived Experience or Public Contribution:**

This manuscript was conceptualised and led by two researchers in designated lived experience research roles. These researchers identify as having lived/living experience of mental health challenges, service use and histories of trauma. The partnership between these two lived experience researchers (first and second authors) occurred through their shared interest in lived experience‐led services and service evaluation. These authors use their lived experience to inform and guide this research, including the study's design, analysis and interpretation. The fourth author is also in a designated lived experience researcher role and contributed to the writing and revisions of the paper.

## 1 Introduction

Service users have long advocated for lived experience‐led services [1, 2]. Traditional psychiatric services are often perceived by service users as coercive and harmful [3, 4], fuelling a strong desire for alternative models grounded in lived experience (LE) principles and emphasising autonomy, connection and meaning‐making [5]. LE or peer‐run services are delivered by individuals who have personally faced mental health challenges [6]. As non‐medical services, they often challenge dominant discourses about the origins and treatment of distress [7]. Various models of LE‐run services exist (primarily in the United States and United Kingdom), including peer respites, crisis alternatives, and drop‐in centres [8]. These services employ LE workers to provide responses to distress grounded in relational safety, mutuality, experiential knowledge, sensitivity to trauma, self‐determination and shared power [9, 10].

There are limited LE‐led services in Australia; however, consistent advocacy efforts are driving investment in prioritising the employment of LE workers within services [11, 12]. Despite the increasing employment of LE workers in both clinical and community psychosocial services, this has had minimal impact on the use of outcome measures that focus on measuring clinical symptoms [13, 14]. Recommendations have been made to address the limitations of existing outcome measures in capturing the nuances of peer support, such as increasing self‐agency and personal meaning‐making [15, 16]. Reliance on clinical measures and the limited number of LE‐led services highlight the persistence of clinical centrism, the view that only clinical intervention is truly effective [17, 18].

To date, research on the success of LE services has primarily involved interview‐based qualitative studies, which have demonstrated benefits in reductions in distress, enhanced self‐determination, increased satisfaction, increased hope and reduced harms associated with the traditional mental health system [8, 19, 20, 21, 22]. LE services have also documented tangible measures of impact in reductions in hospitalisations and emergency department presentations [23, 24]. However, less common are outcome measures designed to measure individual change, such as questionnaires that assess personal recovery or quality of life, or experience measures, including quantitative satisfaction or exit surveys [8, 15].

In the absence of measures tailored specifically for LE services, clinical measures are occasionally used to assess the impact of lived experience services; however, these are not designed to capture the more nuanced effects of peer practice [15, 16]. This is because many of these clinical measures (e.g., tools such as the Kessler 10—K‐10, Patient Health Questionnaire 9—PHQ‐9, Generalised Anxiety Disorder 7—GAD‐7) do not reflect factors such as increased social connection and self‐agency, which peer work has been demonstrated to impact [25].

Within National policy in Australia, it has been recognised that service users should be actively involved in developing outcome measures [26, 27]. However, lived experience‐developed outcome measures have moved slowly, with reticence to adopt and see value in alternatives to traditional clinical measures raising concerns about the equitable involvement of lived experience in the development of outcome measures [28].

Exceptions to the use of clinical outcomes include recovery‐oriented outcome tools such as the Recovery Assessment Scale (RAS), which measure personal recovery and well‐being beyond symptom reduction [29, 30]. Unlike clinical measures of symptoms, personal recovery reflects living well and finding meaning and purpose regardless of the presence of mental health symptoms [31]. Recovery and wellbeing scales are often preferred by people with lived experience as they focus on what is more meaningful and are less medicalised and deficit‐focused [32]. Significantly, while recovery‐oriented approaches and the LE workforce are recognised as beneficial, services are still primarily evaluated in terms of clinical outcomes [33].

Therefore, there is a gap between what service users perceive as meaningful outcome measures and the clinical indicators that services and funders often value [34]. A recent example in a co‐delivered crisis service in Australia found that while service users valued a reduction in their distress, they wanted a measure that reflected safety, connection and empowerment resulting from accessing the service [35]. Pointedly, these findings are not specific to LE‐run or even co‐delivered services; research has identified that people accessing therapists value self‐understanding, self‐agency and social connection over measures of symptom reduction [36].

What people accessing services value is also captured through service experience measures that use quantifiable data on what matters in how services are delivered [34]. Historically, outcome measures have been prioritised over experience measures because they are seen as providing a more direct indicator of service efficacy [37]. However, understanding and measuring the experience of service reveals important indicators of whether a service is fit for purpose by assessing qualities such as accessibility, person‐centredness and cultural safety in mental health services [38].

Ultimately, the reliance of funders on clinical measures shapes perceptions of what counts as credible evidence. This presents challenges in evaluating LE‐run services that offer alternatives to biomedical approaches. However, the lack of appropriate evaluations demonstrating service impact limits their expansion. A further challenge to assessing LE‐run services is that reports of their effectiveness are often conflated with lived experience participation in traditional services, rather than recognised as autonomous, LE‐led services [39]. Therefore, the definition of LE‐led services adopted for this review is based on three key characteristics: services are exclusively managed by people with lived experience; services operate independently of traditional clinical systems; and services provide non‐medical mental health support [8, 9, 40]. They may also be referred to in the literature as peer or consumer‐run.

This scoping review aims to examine outcome and experience measures used in LE‐led services to evaluate the viability and acceptability of LE services as alternatives to traditional mental health services. In doing so, the key objective of this review is to highlight the outcome measures used in LE spaces and how they are used to demonstrate impact.

## 2 Method

As the review sought to synthesise outcome measures used across different LE‐run models, a scoping review methodology was chosen for its suitability in identifying and mapping breadth and diversity [41].

### 2.1 Scoping Review Question

The review sought to answer the following question: What are the outcome measures and experience measures used in LE‐led services to assess service impact and service user experiences?

### 2.2 Study Selection

#### 2.2.1 Inclusion and Exclusion Criteria

The Joanna Briggs Institute (JBI) population, concept and context (PCC) framework for scoping reviews [42] was used to define the inclusion criteria and to select studies aligned with the review's objectives (see Table 1). The PCC framework was selected to inform the search strategy to keep the scope broad rather than focused on clinical or intervention comparisons [43]. Population or participants (P) is the specific group of people being studied, concept (C) refers to the core idea being studied, and context (C) is the setting under investigation [42].

**Table 1:** Inclusion criteria for scoping review according to population, concept, and context framework.

PCC element	Definition
Participants	Literature on services led and delivered by people with LE of mental health challenges. Includes peer, service user, and consumer‐operated services.
Concept	Studies that include the implementation of outcome or experience measures, such as surveys and feedback tools, but exclude interview‐only evaluations.
Context	The literature was restricted to services that were not part of clinical services and were not co‐delivered by traditional mental health professionals (such as programmes in which peers worked alongside clinical staff – e.g., mental health practitioners, psychologists, nurses, social workers).

Inclusion and exclusion criteria were aligned with the definition of LE‐led mental health services, which have three key characteristics: services are led exclusively by people with lived experience; services operate independently of traditional clinical systems; and services provide non‐medical mental health support. [9]; (Grey and O'Hagan, 2015 [40]). The review included empirical studies that: (1) were published in English; (2) included an outcome measure OR an experience measure; (3) the measure is a quantifiable tool; (4) the outcome/experience measure allows for comparison between individuals on the impact of the service on their mental health, wellbeing or recovery OR their experience of a service; (5) service reported in the research is delivered by peer or LE workers with their own first‐hand experience of a mental health challenge; (6) service has a mental health support focus (in contrast to providing education); (7) the tool/experience measure needs to have been used in a service; (8) service was delivered by paid peer/LE workers (not volunteers) to recognise the important differences in formality and responsibility between volunteer and paid roles.

Studies were excluded if: (1) there were duplicates; (2) written in language other than English; (3) the evaluation of impact of a service on mental health or consumer experience/satisfaction was exploratory or descriptive: for example, a focus group or interview, which does not allow for comparison of individuals; (4) there were no reported outcome/experience measures; (5) service was run by volunteers; (6) services co‐delivered by peers and clinicians and/or (7) service is attached to a clinical service. The rationale for exclusion criteria 5, 6 and 7 was that the focus was to consider services that provided a unique and viable alternative to existing clinical services.

As this scoping review focused on identifying outcome measures used across lived experience services, the search was limited to primary research articles; conference abstracts, theses, dissertations and grey literature were therefore excluded.

### 2.3 Search Strategy

Preliminary scoping searches were conducted to identify keywords and refine the search strategy. Four health/psychology bibliographic databases (PsycINFO, Ovid Medline, CINAHL and Embase) were searched for all relevant literature available up to the date of the search (January 22, 2026).

The search strategy was developed using a combination of established search terms for lived experience, service types and outcome measures to identify research that aligned with the population, concept and context framework (see Table 2). Boolean operators were used to maximise the sensitivity and precision of the terms used.

**Table 2:** Search terms used in the scoping review, aligned with population, concept, and context framework.

PCC element	Search terms
Participants	“Lived experience” OR “peer run” OR “consumer run” OR “peer‐led” OR “consumer‐led” OR “peer‐operated” OR “consumer‐operated” OR “consumer led” OR “peer led OR “peer operated” OR “consumer operated” OR “peer‐run” OR “consumer‐run” OR “service user led” OR “service user run”
(AND) Concept	“Outcome measure” OR “impact measure” OR “experience measure” OR evaluation OR “outcome tool” OR “impact tool.”
(AND) Context	“crisis café” OR “crisis alternative” OR “peer respite” OR “alternative to hospital” OR “mental health” OR “crisis space” OR “safe space*” OR “safe haven” OR warmline OR “warm line” OR “peer service” OR “lived experience service” OR “consumer service” OR “service user service”

Electronic search results were exported to COVIDENCE (a web‐based software platform that streamlines the review process) for evidence screening and review. The first author initially screened all titles and abstracts independently against the inclusion and exclusion criteria. The full texts of the included articles were subsequently screened by the second author, and discrepancies were resolved through discussion between the two authors until consensus was reached on whether the papers met the search criteria.

#### 2.3.1 Data Extraction

The second author extracted relevant information from the studies and entered it into an Excel spreadsheet (see Appendix A). The data included methodological features (e.g., type of service, study design, sample size, outcome measures used and outcomes/changes identified). The first author undertook a final quality check of the data extraction. As is common in scoping reviews, an appraisal of study quality was not conducted [43].

#### 2.3.2 Data Analysis

Data analysis compared information within each extraction category, highlighting commonalities and variations. The research questions provided a framework for analysing the data, with the analysis focused on identifying the use and types of outcome and experience measures used across the literature. The first and second authors then explored relationships, similarities and differences in the data [44]. Both researchers discussed emerging findings until a consensus was reached. The entire research team then reviewed these findings to ensure consensus.

## 3 Results

**Figure 1:**
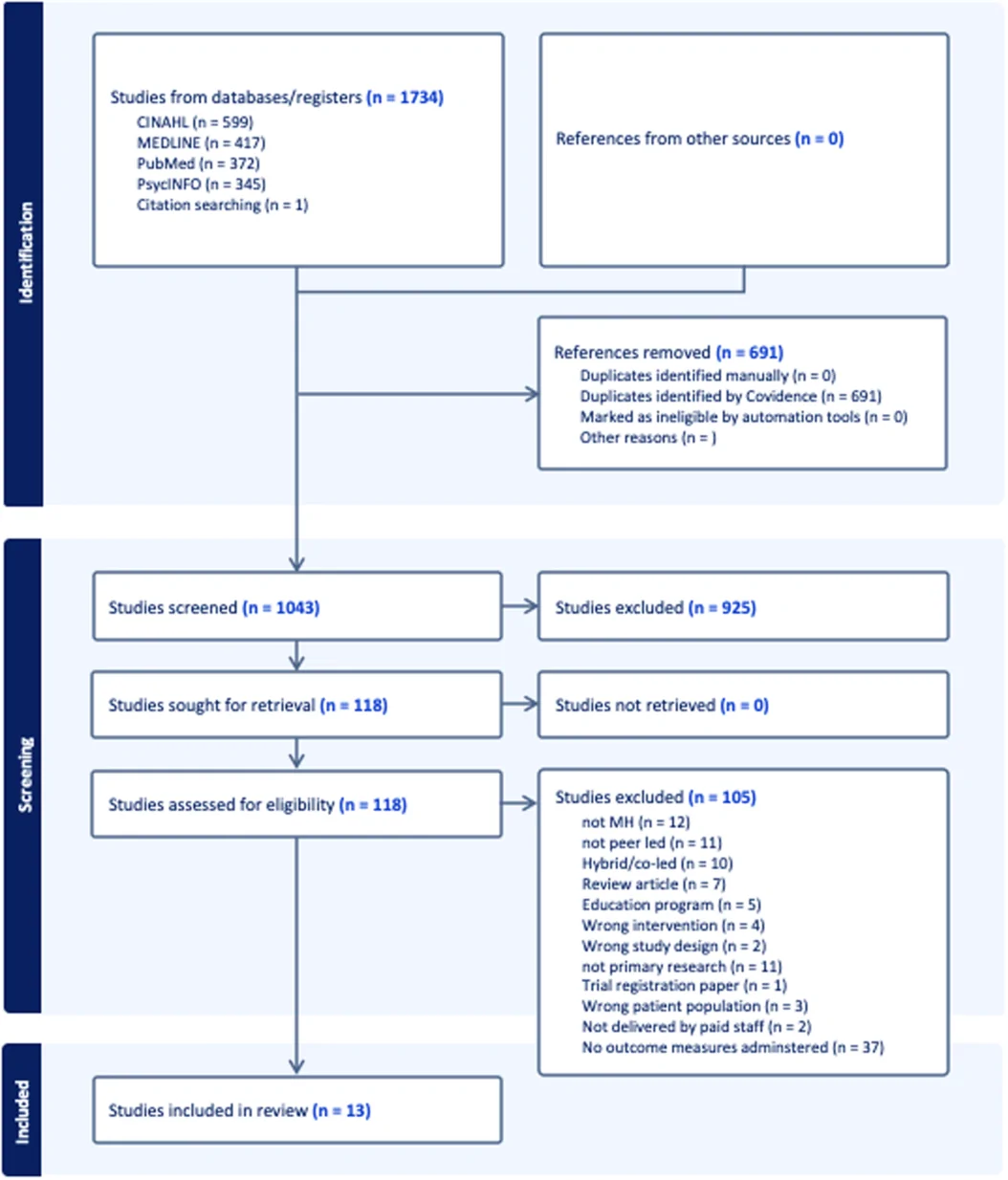
Prisma diagram.

See Figure 1 below for the outline of the results in the PRISMA flow diagram.

After locating 1734 studies, 691 duplicates were removed by Covidence. The titles and abstracts of the remaining 1043 articles were then screened, and 925 studies were deemed irrelevant. The full texts of 118 studies were screened for eligibility, and 105 were excluded. Data were then extracted from the 13 included articles.

### 3.1 Description of Studies

Included studies were published between 1993 and 2025, with most published in the last 20 years and about half (6) published in the last 10 years. Most articles were published in the United States (*n* = 9).

#### 3.1.1 Types of LE Run Services

A range of different services were featured in the studies, as depicted in Table 3 below.

**Table 3:** Types of LE run services.

Type of service	Study reporting on service
Consumer operated drop‐in centre	[45]
Peer warm line	[46]
Peer‐run hospital diversion programme	[47]
Residential respite programme	[24]
Peer‐led workplace suicide prevention programme	[48]
Art‐based programme	[49]
Peer‐led community‐based treatment	[50]
Self‐help agencies	[51, 52]
Mental wellness intervention	[53]
Evaluations that covered a range of consumer‐run services	[52, 54, 55]

### 3.2 Study Design

The studies included in this scoping review also had a range of designs, with mixed methods and randomised controlled studies being the most common (see Table 4), and sample sizes ranging from 31 [49] to 3759 [48].

**Table 4:** Study designs.

Study design	Publication
Mixed methods studies	[49, 50, 51]
Qualitative‐structured interviews (measures implemented during interviews)	[45, 54]
Comparative designs (comparing peer‐run services with traditional services)	[47] (uncontrolled, single group retrospective study design) [48]; (exit surveys)
Randomised controlled studies	[52, 53, 55, 56]
Propensity score matching (comparing respite programme users vs. non‐users)	[24]
Longitudinal study	[46]

#### 3.2.1 Outcome and Experience Measures Used in LE‐Run Services

In addition to outcome measures, demographic variables (such as age, gender), status variables (such as employment, finances, housing), and diagnoses were sometimes collected. Outcome measures included subjective indicators of personal recovery and wellbeing, clinical measures of symptom severity and rates of hospital readmission. Experience measures included indicators of service experience and participation levels. In many instances, a combination of measures was used. The types of tools included psychometrically tested/validated questionnaires, purposefully designed surveys and questionnaires, and adapted versions of existing tools/measures, as presented in Table 5.

**Table 5:** Types of survey/tools and what they measure.

Type of survey or tool	What is being measured
Psychometrically tested questionnaires	
Making Decisions Empowerment Scale [55]	Personal empowerment and its effect on personal and social functioning
Common Assessment Protocol [55]	Demographic variables, status variables (e.g., employment, finances, housing), clinical variables (e.g., symptoms, substance use), and subjective indicators (e.g., quality of life, hope, recovery, and social inclusion)
DERS—Difficulties in Emotion Regulation Scale [49]	Emotional regulation
RAS—Recovery Assessment Scale [46]	Recovery impact of service
Quality of life scale (QOL) [47, 54]	Quality of life
Symptom Checklist (SCL‐10) [54]	Clinical symptoms
Patient Health Questionnaire‐9 Generalised Anxiety Disorder‐7 Perceived Stress Scale [53]	Clinical symptoms
Purposely designed surveys and questionnaires	
4‐item satisfaction measures [45]	Members’ perceptions and evaluation of the service
9‐item quality of participation tool [54]	Perceptions of involvement in the consumer‐run service
Interview schedule measuring member participation in consumer‐run services [54].	Quality of life, social connection, and employment/education
Purposely adapted versions of existing tools/measures	
Five‐part, 70‐item survey based on the service type and quality of care standards, New York State Mental Hygiene Law, 2010 [47]	Evaluating the experience of environment, services and staff, impact on recovery and life satisfaction
19‐item survey adapted from the QOL Index version III [47]	Life satisfaction
6‐item exit survey, including involvement in decision making [48]	Empowerment
Four items from Maton's meaningful activity scale (1990) and six items about community integration [55]	Community integration
Ten items from Rosenberg's (1965) self‐esteem scale [55]	Personal empowerment
Seven items for the social provisions scale, plus one additional item to measure perceptions of support [55]	Social support
Comparison of data between groups	
Comparison data between the lived experience‐led service and treatment in a hospital [50]	Re‐admission rates (3 and 12 months), and factors impacting service user experience
Comparison of empowerment measures between consumers using consumer‐operated service and traditional MH services only [55]	Empowerment
Compared consumer‐run services with assertive community treatment (ACT) [54]	Consumer‐run service members and ACT users’ differences in terms of demographics, SES, and clinical features that may affect functioning (e.g., diagnoses, use of psychotropic medications)
Experience of service	
Compare across programmes‐ interactions with staff, physical environment, and support (Ferrans & Powers, 1998)	Service experience, including the role of staff
Recommendation to others [48, 55]	Service satisfaction
6‐question exit survey [48]	Acceptability of the programme

#### 3.2.2 Method/When Measures Were Used

The tools were used at various points throughout the service experience. Many studies collected data at baseline from consumers who had recently engaged with a service, and then at periodic intervals during their participation in the service [24, 46, 49, 52, 54, 55, 56]. One study [51] administered one‐off surveys, while Doran and colleagues [48] used information from a database in addition to an exit survey. Another study asked consumers to complete a retrospective assessment following their service experience [47].

### 3.3 Findings Reported on Outcomes by LE Services

As indicated in Table 5 (above), the studies assessed a wide range of concepts related to outcomes and service user experience. Measures of personal recovery (reported in seven studies) showed increases in most studies, with effects on meaning‐making, empowerment, and self‐agency, while one study reported no significant change in RAS scores [46]. The complexity of personal recovery and the limited contact provided by peer warm lines may be critical in understanding this variation in results. An increase in empowerment over time was observed across a variety of lived experience‐run services, with significant but small differences among drop‐in, mentoring, and advocacy services [55].

Life satisfaction and social connection scores were higher, and less stigma was reported in a peer‐run service compared with a non‐peer‐run acute service [47]. Similarly, Nelson and Lomotey [54] identified improved social outcomes related to social support, quality of life and community activities. They concluded that quality, rather than the frequency of attendance, was important and increased service satisfaction [54]. Overall, satisfaction and positive service experience measures were higher in LE‐run services than in traditional non‐LE services [45, 47, 49]. Greater satisfaction was associated with the ability to influence change and more autonomy [45]. Consumers also valued the physical environment, which was viewed as offering more privacy and greater personal agency over how they spend their day than the non‐LE‐run service [47].

While one study comparing LE and traditional acute mental health services found that clinical measures and socio‐demographic characteristics were similar across LE and traditional clinical services [55], differences in gender, culture, diagnosis and socio‐economic status were noted in Manuel et al.'s study [50]. In this study, people who accessed the LE‐led service were less likely to come from lower socio‐economic areas, and less likely to be diagnosed with psychotic, bipolar, substance use and personality disorders than those accessing the acute service. Another difference between a LE and a traditional acute unit was longer stays in the peer service compared with traditional mental health services. Although Manuel and colleagues did not explore the potential reasons why individuals stayed longer, they did report lower hospital readmission rates [50]. Similarly, another study reported value in lived‐experience‐run services diverting individuals in crisis away from the ED [48].

## 4 Discussion

This scoping review reported on outcome and experience measures used in LE‐led services. Studies used diverse outcome measures to demonstrate their impact, including rigorous randomised controlled trials. Findings from this scoping review provide evidence of impact across LE‐led services, indicating that these services deliver effective support. While studies highlight the effectiveness of LE‐led services, the dominance of clinical priorities often sidelines this evidence and the impacts of LE‐led services [18].

Many of the outcome measures used in LE spaces were psychometric tools for examining consumers’ mental health, with some services measuring clinical symptoms [49, 53]. These outcome measures likely reflect an attempt to provide evidence for LE services in a manner comparable to that for non‐LE services, thereby demonstrating that they remain a viable alternative. Similarly, to provide evidence of the value of the service, some LE‐led services collected data on hospital readmission and emergency department presentations [24]. However, these measures may not capture the outcomes that matter to consumers or reflect the unique contributions of lived‐experience approaches [16]. In a consumer‐led study by Waks and colleagues [57] into what matters most to consumers, the authors found that consumers seek outcomes such as self‐determination, empowerment, social connectedness and quality of life that align with personal recovery, rather than purely clinical measures.

Likewise, preferences for outcome measurement in co‐delivered and clinical services have identified the need to include qualities associated with personal recovery, such as personal agency and social connection [35, 36, 58]. Several studies included in the review used outcome measures to assess personal recovery; for example, Dalgin and colleagues used the Recovery Assessment Scale [46]. Tools such as the RAS are increasingly recognised in the psychosocial sector and have been adopted as useful in measuring what matters to consumers [59]. Findings also identified a gap in outcome measures that tap into changes in social determinants of health, which are increasingly recognised as critical to mental health and associated with increased service use [60, 61].

Findings reveal that measures of service experience, which assess the quality of support provided by a service, are not commonly implemented. These experience measures are distinct from measures of individual change [62]. Understanding the quality of the relationship with staff and the experience of receiving services is important for understanding the link between the service experience and outcomes related to personal recovery. Many LE services identified increases in empowerment, which may be related to the connectedness and safety people feel when accessing these services and may prove to be important mechanisms of change.

People with lived experience have been recognised as needing to be the cornerstone of the development of outcome measures [63]. However, this review shows clinical outcomes continue to dominate even in LE‐led services. Across the studies, there was also a notable gap in the development of LE outcome measures. The findings reinforce the need for further research into what matters most to consumers when measuring the impact and experience of services, with the priorities of people with LE largely unheard [64]. It is also unclear from the findings whether consumers have different expectations about how their experience with LE‐led services may be measured compared with clinical or psychosocial services.

While not specific to LE‐led services, robust literature shows that consumers prefer outcome measures that emphasise autonomy, personal meaning‐making and social connection [35, 36, 58, 65]. These qualities align with lived experience values and peer practices, providing a starting point for future research into preferred outcome measures in lived experience‐run services. Given that these outcomes are valued across settings, this supports the use of non‐clinical outcomes more broadly. Overall, the findings of this scoping review highlight the need to carefully consider outcome measures that are congruent with lived experience practices and that centre the needs and preferences of the individuals who access these services.

This review was limited to lived experience‐led services and may have missed important contributions of lived experience outcome measures used in lived experience programmes within clinical or community mental health services. Other limitations include a broad mapping of existing literature rather than detailed exploration of service types and findings. The review summarises findings from the studies but does not include a critical appraisal of these findings. Future research may explore outcome measures designed by people with lived experience and used more broadly. Further exploration is needed to understand how outcome measures in LE‐run services are chosen, and the involvement of consumers in deciding which outcomes services measure.

## 5 Conclusion

This scoping review found that LE‐run services use a range of outcome and experience measures to assess the impact and service experience. The results indicate that a limited number of measures developed by people with lived experience are used in lived experience‐led services and suggest a pull towards services using existing psychometric tools to compare and demonstrate evidence in relation to more traditional clinical services. There was a wide range of reasons for collecting measures, and many services used a mix of outcome and experience measures. As the outcome measures currently used in LE‐run services were not designed by people with LE, they may not align with lived experience practice and may not capture what is meaningful to service users in terms of personal recovery, wellbeing and healing. This review highlights the need for lived experience‐led services to align outcome measures with the values of LE practice and what is important and meaningful to consumers.

## Author Contributions

**Helena Roennfeldt:** conceptualisation, writing – original draft, writing – review and editing, methodology. **Heather Craig:** conceptualisation, writing – original draft, methodology, writing – review and editing. **Edith Botchway, Richard Schweizer, Christina Ng, Laura Hayes:** writing – review and editing.

## Funding

The authors have nothing to report.

## Conflicts of Interest

The authors declare no conflicts of interest.

## Data Availability

The data that support the findings of this study are available on request from the corresponding author.

## Appendix A

### A.1

See Table A1.

**Table A1:** Characteristics of the included studies.

Author, year	Description of service	Aim/purpose of outcome/experience measures	Data source/tool used	Sample size	Country	Study Design	Results
[47]	Homelike residential setting in New York ‐ a hospital diversion programme where guests are assisted to cope with psychological distress.	To examine consumer perceptions and experiences with services compared to an acute (non‐peer) inpatient psychiatric programme.	A 5‐part, 70‐item survey, including 19 items adapted from the Quality of Life Index, version III.	39 adults	USA	Uncontrolled, single‐group retrospective design	Consumers received more of what they wanted from staff, services, and the diversion programme environment. Results showed that the programme is an effective alternative for clients who are in a position to make an informed choice during a crisis.
[24]	Short‐term peer‐led residential support for guests in a homelike setting, for up to 14 days per visit.	To establish inpatient and emergency service use, including crisis support services, crisis residential services, and short‐ and long‐term psychiatric inpatient hospitalisation for guests	Demographics and service use data, including clinical characteristics (including Global Assessment of Functioning)	139 matched pairs of adults	USA	Propensity score matching	The probability of using inpatient/emergency services was approximately 70% lower for respite guests compared with matched nonusers of the respite programme, though the association was complex and non‐linear.
[46]	Peer‐run “warm line,” staffed by peer specialists trained in Intentional Peer Support; designed to be a mutual conversation focused on moving forward in recovery.	To assess impact of warm line as part of recovery process.	Recovery Assessment Scale (RAS), community integration measures, crisis service usage.	48 adults	USA	Survey	Although warm line callers did not show statistically significant differences in RAS scores (which might be expected – change in RAS would be unlikely based on a phone call intervention), there were some increases in measures of community integration. Callers were more optimistic about the future and believed personal goals were achievable.
[48]	MATES: Multimodal, peer‐led workplace suicide prevention programme for individuals in the construction industry in Australia.	To quantify service demand, determine demographics, occupational profile, presenting issues, referral pathways, and perceived benefit of case management among service users.	Routinely collected administrative data, self‐report exit survey to obtain information about perceived benefits	3759 adults (including 14 exit surveys)	Australia	Exit survey and analysis of routinely collected data	All respondents to the exit survey agreed that their concerns were addressed during case management. The study demonstrated the acceptability of a non‐clinical, peer‐based case‐management model, nested within a referral pathway that does not require gatekeeping by a health professional.
[49]	A peer‐led online, art‐based skill programme held weekly for 2 h over an 18‐week period, for people living with borderline personality disorder.	Assess acceptability of the programme, its role in ameliorating symptoms, and evaluate if the programme improved emotion regulation	Questionnaire, including the Difficulties in Emotion Regulation Scale (DERS) and qualitative feedback questions	31 adults	Australia	Repeated mixed methods design	No significant difference in baseline DERS for those who completed vs. those who did not complete the programme. There was a significant improvement in emotion regulation across all domains following the programme, with a very large effect size. Participants stated that having a facilitator with LE impacted their decision to stay in the programme. Participants valued connecting with others, building an improved understanding of the self, and having greater hope for living well.
[50]	Peer‐led 7‐bed acute unit, in a homelike environment based in an urban city in NZ.	To compare sociodemographic and admission characteristics of peer‐led service compared with acute inpatient hospital, to understand the lived experience of the service and sociocultural factors impacting service delivery.	Routinely collected administrative data collected by peer‐led service and comparison (regional public hospital, specialised MH acute inpatient unit), semi‐structured interviews with guests, peer specialists, and clinical staff.	Quantitative = 1770 adults; Qualitative 23 adults	New Zealand	Mixed methods process evaluation	Guests were unequivocally positive about their experiences of the support they received and of the peer‐led unit. Just under one‐third of the guests were admitted to the traditional acute hospital within three months of discharge from the peer‐led unit.
[45]	6 consumer‐operated drop‐in centres, each established for at least 2 years, which provide an alternative or adjunct to traditional MH services.	To survey individual consumers’ perceptions and evaluations of the centre	Interview instrument, including adapted Group Environment Scale (GES), Community Oriented Programmes Environment Scale (COPES), Client Satisfaction Questionnaire (CSQ‐8)	120 adults	USA	Structured interview	Almost all members were satisfied and believed the centres fulfilled most of their social needs. Satisfaction is largely the result of the positive social environment. An overwhelming majority of respondents reported that the centres had positive effects on their lives.
[54]	4 consumer‐run organisations (Consumer/survivor initiatives ‐ CSIs) in Ontario, each unique, providing opportunities including self‐help groups, drop‐in and 1:1 peer support, and a ceramics collective	To evaluate the impact of CSIs on individual members and social systems	Structured interviews, including adapted Levy's 9‐item measure assessing perception of involvement in the CSI; adapted Social Provisions Scale; Rosenberg ‘s self‐esteem scale; Quality of Life; Symptom checklist (SCL‐10)	79 adults	Canada	Structured interview)	Quality of CSI participation was positively related to socially oriented outcomes ‐ social support, quality of life, and community integration. New members were more likely to participate in internal activities.
[55]	Consumer Operated Service Programmes (COSPs) across 8 sites ‐ safe and welcoming peer‐led environments offering opportunities to interact with other peers and connect to the wider community.	To determine whether significant differences in empowerment existed between those who attended a COSP in addition to traditional MH services and those who were not exposed to such peer‐led programmes.	Common Assessment Protocol, Making Decisions Empowerment (MDE Scale), Personal Empowerment Scale (PE), Organisationally Mediated Empowerment Scale (OME)	1827 adults	USA	RCT	COSPs in general have a positive additive effect on empowerment when measured subjectively (MDE, PE). There was little evidence that the programmes affected organisational empowerment, though a longer observation period (> 12 months) may have allowed an effect to be seen.
[51]	Self‐help agencies (SHAs) in the US ‐ programmes that are planned and operated by consumers.	To hear consumer's perceptions of SHAs and explore whether SHA and traditionally run community mental health agency (CMHA) consumers differ in their programme perceptions.	Survey, including Community Oriented Programme Environment Scale (COPES)	1278 adults	USA	Mixed methods	Although self‐help leader aspirations for programme environments and consumer perceptions were significantly different, both emphasised involvement, support, and autonomy. SHAs were found to perform at least as well as traditional MH services. SHAs were perceived to be below the average CMHA in terms of order and organisation.
[56]	Self‐help agencies (SHAs) located in close proximity, that are peer‐led drop‐in centres that provide social support, material assistance, and vocational opportunities, in a nonthreatening environment.	To determine the effectiveness of combined SHA and community mental health agency (CMHA) services in assisting recovery.	Interviews, including Personal Empowerment Scale, Self‐efficacy Scale, Independent Social Integration Scale, Brief Psychiatric Rating Scale, Hopelessness Scale	505 adults	USA	RCT	Combined SHA and CMHA services were significantly more effective at promoting recovery than CMHA services alone.
[52]	A consumer operated service programme (COSP) that includes peer support groups, material resources, drop‐in socialisation, and direct services such as provision of information or referral.	Conducting a two‐group RCT to determine effectiveness of combined COSP and CMHA, vs. CMHA only services	Personal Empowerment Scale, Self‐efficacy Scale, Independent Social Integration Scale, Brief Psychiatric Rating Scale, Hopelessness Scale	139 adults	USA	RCT	The joint services did not perform better than CMHA alone. Independent social integration, personal empowerment, and self‐efficacy improved to a greater extend in CMHA only condition than in the combined service condition.
[53]	Mood Lifters’ (ML)‐ a mental wellness intervention run by peer leaders in a group setting, addressing common barriers to care, and introducing skills across meetings (each 1 h long)	To test the impact of the ML programme	Patient Health Questionnaire (PHQ‐9), Generalised Anxiety Disorder (GAD‐7), Perceived Stress Scale (PSS)	85 adults	USA	RCT	ML participants had a significant decrease in anxiety, but no meaningful change in depression or perceived stress. There was no difference in treatment delivery between leaders who were peers without clinical experience and professionals (who also had LE).
